# A long-term high-fat diet influences brain damage and is linked to the activation of HIF-1α/AMPK/mTOR/p70S6K signalling

**DOI:** 10.3389/fnins.2022.978431

**Published:** 2022-09-16

**Authors:** Fei Chen, Wen-min Yi, Sheng-yuan Wang, Ming-hao Yuan, Jie Wen, Hong-Yan Li, Qian Zou, Shu Liu, Zhi-you Cai

**Affiliations:** ^1^Department of Neurology, Chongqing Medical University, Chongqing, China; ^2^Chongqing Institute Green and Intelligent Technology, Chinese Academy of Sciences, Chongqing, China; ^3^Chongqing School, University of Chinese Academy of Sciences, Chongqing, China; ^4^Department of Neurology, Chongqing General Hospital, Chongqing, China; ^5^Chongqing Key Laboratory of Neurodegenerative Diseases, Chongqing, China; ^6^Department of Neurology, Guangdong Medical University, Zhanjiang, Guangdong, China; ^7^Department of Neurology, The Affiliated Hospital of Southwest Medical University, Luzhou, China

**Keywords:** ageing, obesity, high-fat (HF) diet, autophagy, HIF-1α, AMPK/m TOR pathway

## Abstract

High-fat diets (HFDs) are related to the incidence of obesity and diabetes, but the effect of high-fat diet-induced brain damage remains to be clarified. In our study, we found that 24 weeks of a HFD effectively induced obesity and a change in fur color in mice. In addition, the mice also exhibited deficits in learning and memory. We further found that autophagic flux was impaired in mice after HFD feeding. Hypoxia-inducible factor 1α (HIF-1α) expression was significantly increased in HFD-fed mice, and HFD feeding inhibited adenosine monophosphate-activated protein kinase (AMPK) phosphorylation and induced mechanistic target of rapamycin (mTOR) phosphorylation and p70S6K expression. Treatment of HFD-induced BV2 cell model with palmitic acid (PA) was used to further verify a similar result. We concluded that improving tissue hypoxia or enhancing autophagy through the AMPK/mTOR/p70S6K pathway may be a relevant strategy for improving obesity- and ageing-related disorders.

## Introduction

With the increase in restaurant businesses and takeaway food outlets, the global epidemic of HFD structures continues unabated Prentice and Jebb (2003). It is clear that the human health span and life span have increases over the past three decades. However, prolonged overconsumption of a HFD induces obesity and may counteract the health benefits of modern civilization. Excessive fat intake makes people more susceptible to obesity. Obesity in middle age significantly increases the risks of developing noncommunicable chronic diseases (NCDs), such as coronary artery diseases, cerebrovascular accidents, type-2 diabetes mellitus, dementia, hyperlipidaemia, Parkinson’s disease (PD) and Alzheimer’s disease (AD), later in life Knight et al. (2014) and Abbott et al. (2019). As a major manifestation of metabolic syndrome, obesity not only impairs aesthetic appearance but is also accompanied by low-grade systemic inflammation and elevated blood pressure, supraphysiological fasting plasma glucose, high serum triglycerides, and low high-density lipoprotein levels Hoffman et al. (2015). To date, there have been many epidemiological surveys conducted on abnormal lipid metabolism with cognitive impairment worldwide, but there have been few reports on the molecular pathology of obesity-related brain damage.

Accumulating evidence indicates that the accumulation of senescent cells in the brain can lead to disease pathology (Sikora et al., 2021). According to a previous report, excess adiposity is associated with local tissue hypoxia Hosogai et al. (2007). The cellular response to hypoxia is coordinated by HIF-1α, a heterodimeric transcription factor thought to be a key regulator of the host response to hypoxia. HIF-1α expression levels are increased in a hypoxic tissue microenvironment. Hypoxic stimuli increase HIF-1α protein levels by inhibiting its degradation by the proteasome. AMPK is a highly evolutionarily conserved metabolic regulator that maintains energy homeostasis during metabolic stress Hardie (2015). The enzyme AMPK is a heterotrimeric protein composed of a catalytic α-subunit and regulatory β- and γ-subunits. The activation of AMPK requires the phosphorylation at threonine 172 of the α catalytic subunit by upstream kinases. AMPK plays a vital role in regulating obesity Carling et al. (2008). The mechanistic target of rapamycin (mTOR), a protein kinase, regulates mammalian metabolism and physiology especially in cell survival, protein synthesis and autophagy. mTOR exists in two multiprotein protein complexes, –mTORC1 and mTORC2. Rapamycin-sensitive mTORC1 regulates protein synthesis and cell growth through the phosphorylation of p70 ribosomal S6 kinase 1 (p70S6K1: Thr389). Mammalian target of rapamycin complex 1 (mTORC1) can promote many anabolic processes, including the biosynthesis of proteins, lipids and organelles, and by limiting catabolic processes such as autophagy and apoptosis. According to previous reports, under physiological conditions, mTOR activation inhibits autophagy Wang and Zhang (2019). Moreover, as a classically regulated autophagy signaling factor, mTOR kinase activity may be suppressed by the phosphorylation of AMPK.

Herein, we hypothesized that diet-induced brain impairment may induce cellular senescence through the activation of AMPK and mTOR signaling and is involved in changes in autophagy in progressive disease occurrence, ultimately resulting in cognitive impairment.

## Materials and methods

### Animals and treatments

All procedures were approved by the Institutional Animal Care and Use Committee of Chongqing Medical University. Studies were conducted in accordance with institutional guidelines for the care and use of laboratory animals.

Male C57BL/6 mice (13 months old, weighing 25–33 g) were obtained from Chengdu Dossy Experimental Animals Co., Ltd. (Chengdu, China). The mice were maintained in individual cages under controlled light and environmental conditions (12 h light/12 h dark cycle at 23 ± 2°C and 50 ± 10% humidity). After 1 week of adaptation, the mice were randomly divided into one of two groups, balanced by weight, and the mice were fed a regular chow diet (10% kcal fat), referred to as a low-fat diet (LFD) or a HFD (60% kcal fat; D12492; Research Diets, New Brunswick, NJ, USA) Bartels et al. (2009) *ad libitum* for 24 weeks. The general condition of the mice was observed daily, including fur color, mental state, food intake and body weight, and these indices were examined every week. At the end of the study, the mice (18 months old) were tested in the Morris water maze (MWM) task and then were sacrificed by decapitation or deeply anaesthetized with isoflurane. After the mice were perfused with phosphate-buffered saline, the brain was immediately removed, and the tissues were stored at –80°C or 4°C until use.

### Cell culture and treatments

BV2 cells were stored in our laboratory. BV2 cells were maintained in DMEM/F12 culture medium (Gibco Life Technologies, Carlsbad, CA, USA) supplemented with 10% fetal bovine serum (FBS, 187 Invigentech, Irvine, CA, USA) and antibiotics (100 U/ml penicillin and 100 μg/ml streptomycin) and incubated in a humidified atmosphere with 5% CO_2_ at 37°C. The cells were cultured in a 10-cm Petri dish. The HFD BV2 cell model was established by treating the cells with PA medium (containing 100 mM PA) for 24 h (HFD group). BV2 cells in the control (CON) group were treated with the corresponding concentrations of solvent. PA and its solvent (vehicle) were purchased from Kunchuang Biotechnology (Xi’an, Shanxi, China). The reagents were diluted to working concentrations using growth medium.

### Morris water maze test

To assess the spatial learning and memory abilities of mice, the MWM test, which is a widely accepted paradigm, was performed. MWM training included two phases: the place navigation phase and the spatial probe phase. A clear escape platform (8-cm diameter) was submerged 1 cm below the surface of the pool (1.2 m in diameter and 0.4 m in depth, 25 ± 1°C). Behavioral data of the mice were acquired using ANY-maze software (Stoelting Co., Wood Dale, IL, USA) and a digital video camera installed on the ceiling above the center of the maze. Briefly, the mice were trained for 6 consecutive days, with four trials per day lasting 1 min each to spatially locate the submerged platform. Each trial was initiated by placing the mouse in the water. Upon finding and climbing onto the hidden platform, the animals were given 5 s during which they could rest on it. If a mouse failed to find the submerged platform within the allowed time (60 s), it was placed on the platform manually for 30 s. The average latency to reach the platform, average velocity, and mean distance from the platform were analyzed by comparing the average of four trials across each training day to determine the learning performance. On day 7, the mice were subjected to the probe trial to assess spatial memory. The hidden platform was removed for this trial, and then the mice were placed in the quadrant opposite the target quadrant (the previous location of the hidden platform). The number of entries to the platform and latency to reach the target quadrant were analyzed to assess spatial memory consolidation. The MWM test procedures were monitored with the Morris Image System (Shanghai DOiT Industrial Co., Ltd.) *n* = 20 (HFD), 4 (ND).

### Western blot

The mice (*n* = 4/group) were sacrificed humanely after the behavioral experiment, and then the whole brain was quickly removed and stored at –80°C until use. Total protein was extracted from BV2 cells after the cells were treated with PA medium (100 mM) for 24 h and quantified with a BCA protein quantification kit (Beyotime Biyuntian Biotechnology Co., Ltd., China, Cat). Mouse brain tissue or cell lysates were loaded onto 7, 10, or 12% SDS–PAGE gels for separation, depending upon the molecular weight of the target proteins, and then transferred onto PVDF membranes (Bio-Rad, Hercules, CA, USA). The membrane was blocked at room temperature with 5% skim milk in TBST for 2 h on a shaker prior to being incubated with primary antibodies [HIF-1α (1:1000, Proteintech Cat No. 20960-1-AP), mTOR (1:1000, CST, 2984), p-mTOR (Ser2448) (1:1000, CST, 5536), p70S6 kinase (1:1000, CST, 9202), LC3A/B, Beclin-1, Atg3 212 (1:1000, Autophagy Antibody Sampler Kit, CST, 4445)] at 4°C overnight. The membrane was then washed three times with TBST (10 min). The membrane was then incubated with the appropriate horseradish peroxidase-conjugated secondary antibody against rabbit IgG or mouse IgG. The membrane was washed with TBST (3 × 10 min), and the target protein was exposed with an ECL chemiluminescent system (Tanon-5200Multi, Shanghai, China). The grey value of the target protein band was calculated by ImageJ 1.8.0 software (NIH).

### Immunofluorescence staining

The anaesthetized mice (*n* = 3/group) were intracardially perfused with ice-cold phosphate-buffered saline (PBS), and the tissues were paraffin-embedded and sectioned into 4-μm-thick slices using a Rotary Microtome (HM 340E, 9 Thermo Scientific). Following deparaffinization, antigen retrieval was carried out as recommended by the manufacturers with citrate buffer pH 6.0 in a microwave oven for 5 min under high heat and for 15 min under medium heat. For non-specific binding, the sections were blocked for 30 min with 1.5% normal goat serum in PBS containing 0.5% bovine serum albumin. The sections were incubated overnight at 4°C with HIF-1α (1:300, Proteintech Cat No. 20960-1-AP) primary antibodies. After incubation with HRP-conjugated IgG secondary antibody (1:300, Alexa Fluor 488-conjugated goat anti-rabbit IgG, cat. ZF-0516, ZSGB-BIO, China), nuclei were stained with DAPI (10 μg/ml) (Beyotime, China) for 3 min. Then, the sections were washed three times in PBS, anti-quenching fluorescence mounting medium was added, and the samples were covered with coverslips. The frequency of apoptosis in the brain was determined using an *in situ* cell death detection kit (Beyotime) according to the manufacturer’s protocol. All immunofluorescence slides were viewed, and images were acquired using a NEXCOPE microscope (NE900, USA).

### Transmission electron microscopy

After the MWM test, the mice (*n* = 3/group) were euthanized, and the hippocampal tissues were quickly harvested. The samples were prefixed with a solution of 3% glutaraldehyde for TEM analysis. Then, the tissue was postfixed in 1% osmium tetroxide, dehydrated in acetone, infiltrated in Epox 812 for a long time, and embedded. The semithin sections were stained with methylene blue, and ultrathin sections were cut with a diamond knife and stained with uranyl acetate and lead citrate. The sections were examined with a JEM-1400-FLASH Transmission Electron Microscope.

### SA-β-gal cytochemical staining

Staining for the activity of senescence-associated β-galactosidase (SA-β-gal) was performed using a senescence β-galactosidase commercial assay following the manufacturer’s instructions (Beyotime, C0602). Mice (*n* = 3/group) were euthanized in tricaine anesthetic, embedded in Tissue-Tek O.C.T. compound and frozen at –80°C. Ten-micrometer frozen sections were cut onto slides using a cryostat (Thermo). The slides were allowed to dry, washed in PBS solution for 5 min at room temperature, and then fixed with fixative solution for 30 min. Next, the slides were rinsed with PBS, and then 1 ml of β-galactosidase staining solution was added to each sample. After staining overnight at 37°C, blue senescent staining in the cytoplasm in senescent cells was observed at 200× magnification. To quantify the number of SA-β-gal-positive cells, four random visual fields per section were selected and counted with ImageJ software.

### Immunohistochemistry

Prior to incubation with antibody, deparaffinization with xylene and antigen retrieval were performed in a microwave. Sections were incubated in endogenous peroxidase blocker for 10 min. After washing (PBS 3 × 5 min), goat serum was added and blocked at 37°C for 20 min. Samples were incubated overnight at 4°C with mouse anti-human Aβ1-42 monoclonal antibody (1:300, CST, 14974S). The next day, the sections were reheated for 30 min, and PBS was rinsed (3 × 5 min). Then, the secondary antibody (goat anti-rabbit/mouse IgG) was added and incubated at room temperature for 10 min. After subsequent washing in PBS, the peroxidase-conjugated streptavidin-biotin complex was incubated at 37°C for 10 min. After washing with PBS, antigen-antibody complexes were visualized using the DAB horseradish peroxidase color development kit (Jiangsu Beyotime). The slides were then stained with haematoxylin for 5 min and differentiated with hydrochloric acid alcohol for 2 s. The staining results were analyzed by the image analysis software Proplus 6.0 and photographed by a NEXCOPE microscope *n* = 3/group.

### Click-it TUNEL alexa fluor 488 imaging assay

We used the terminal deoxynucleotidyl transferase dUTP nick end labeling (TUNEL) staining method to detect apoptosis by using an *in situ* cell death detection kit (Jiangsu Beyotime). For *in situ* labeling, brain tissue slices were placed in 1 × PBS for 10 min after dehydration. The samples were incubated in the presence of 50 ng/μl proteinase K for 30 min followed by 50 μl of Cytonin™ for 120 min. The samples treated with TACS nuclease were used as the positive control. TUNEL-positive cells were detected with a fluorescence microscope (NEXCOPE, NE900, USA). The percentage (%) of apoptotic cells in the TUNEL assay was obtained by dividing the number of apoptotic cells (TUNEL-positive cells) from the number of total cells (DAPI nuclear staining) in the microscopic field (*n* = 3/group).

### Statistical analysis

Mouse randomization was based on the random number generator function (RANDBETWEEN) in Microsoft Excel. The data were analyzed by GraphPad Prism Software version 8.3.0 (GraphPad Software, San Diego, CA, USA). One-way analyses of variance (ANOVA) and independent-sample *t*-tests were used to compare the differences in measurement data between two groups. The data are expressed as the means ± standard errors of the means (SEM), and differences were considered statistically significant at *P < 0.05.

## Results

### A high-fat diet induced hippocampal-related behavioral changes in aged C57BL/6 mice

**FIGURE 1:**
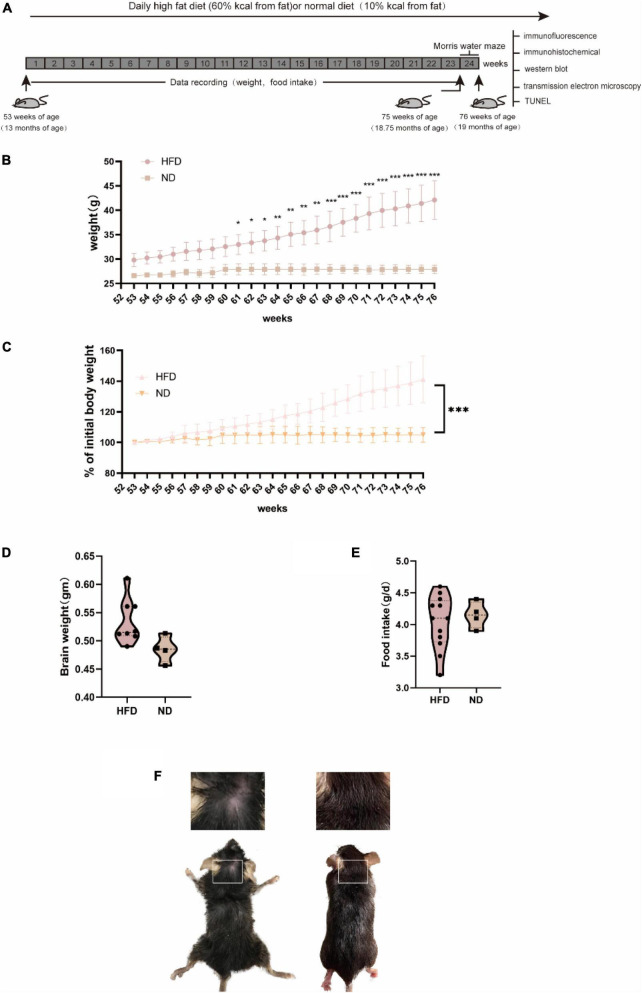
General characteristics of C57BL/6 mice fed with normal diet and high-fat diet. C57BL/6 mice (13 months old) were fed a HFD or ND for 24 weeks. **(A)** Experiment process overview. **(B)** Changes in body weight over time and body weight were significantly increased after 8 weeks of HFD. **(C)** A percentage of the initial body weight during the course of the experiment. Body weight percentage was calculated as (actual body weight/initial body weight) × 100. **(D)** Alterations in food intake were not significantly different between groups. **(E)** (HFD, *n* = 20 mice; ND, *n* = 10 mice) **(E)** Brain weight did not differ between groups. **(F)** At the late stage, the HFD group exhibited an extremely poor status with increasingly sparse hair.

**FIGURE 2:**
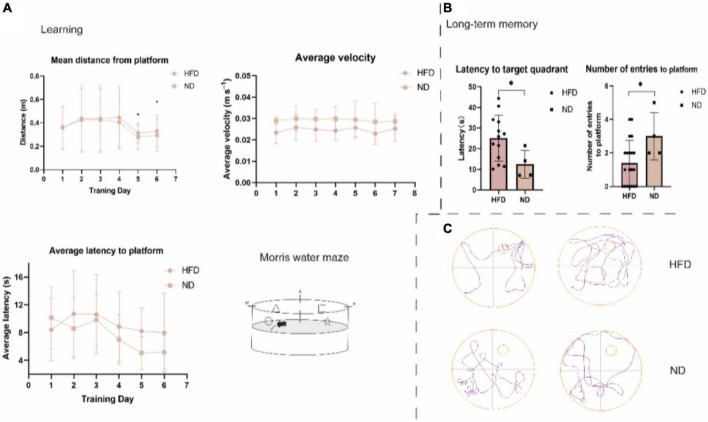
High-fat diet impairs spatial learning and memory in aged C57BL/6 mice. Long-term spatial memory assessed *via* MWM. **(A)** Learning ability. HFD significantly impacted the mean distance from the platform. ND mice spent more time closer to the platform than HFD mice. HFD mice were significantly different from ND mice on days 5 and 6 (*P* = 0.047, *P* = 0.025, respectively). There were no overall differences in average latency to platform or average velocity. *n* = 20 (HFD), 4 (ND), **(B)** Long-term memory. The probe trial revealed an age-related impairment in latency to target quadrant and number of entries to platform, wherein HFD mice were significantly slower to the target quadrant compared to ND mice (*P* = 0.049, *P* = 0.023, respectively). Swimming traces of mice in the probe trial are presented in panel **(C).** p, two-sample *t*-test. Mean ± SEM, *p < 0.05 compared with the ND group, HFD, high-fat diet; ND, normal diet.

We fed 13-month-old male C57BL/6 mice a normal chow diet or a HFD for 24 weeks (6 months) and monitored body weight and food intake (Figure 1). The body weight of mice in the HFD group was significantly lower than those in the control group at week 61 (*P* < 0.05). Moreover, we observed an apparent progressive thinning of body hair, which had a lusterless and sharp appearance in HFD animals. However, the results showed no significant difference in brain weight and food intake between the two groups during the 6 months. Changes in diet have been widely linked to changes in behavior, including hippocampal-dependent cognition, which declines during ageing Daub et al. (2017). Thus, we performed the MWM test to examine whether a high-fat diet damaged brain health and hippocampus-associated behavioral abnormalities in aged mice. Previous research has shown that ageing is linked to impaired learning ability and an increase in the latency to enter the target platform zone. Compared with the ND group, the HFD group showed a significant difference in the number of entries to the target platform. The swim average velocity and average latency to reach the platform in the visible platform trials were similar in the two groups. Aged HFD-fed mice showed increases in the mean distance from the platform on training days 5 and 6. Overall, these data suggest that long-term exposure to a HFD exacerbated the impaired spatial learning and memory in naturally aged mice (Figure 2).

### A high-fat diet increased tissue hypoxia and HIF-1α levels

**FIGURE 3:**
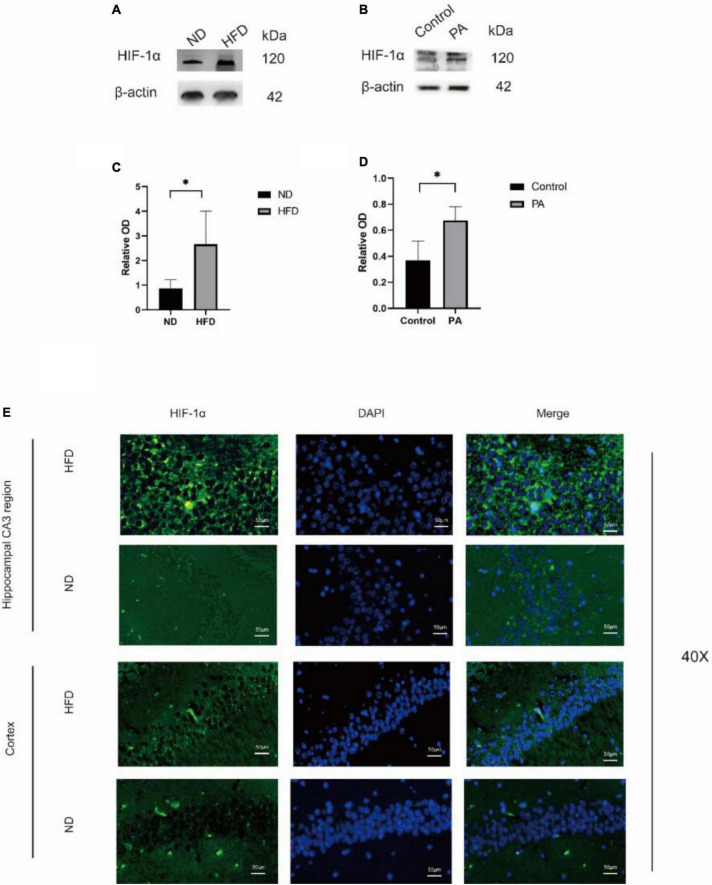
The expression of HIF-1α was observed in hippocampus of mice. Western blot results showing that high-fat diet upregulates the expression of HIF-1α in the hippocampus of aged brain C57BL/6 mice (*n* = 4/group, two-sample *t*-test) **(A,B)**. **(C,D)**, Graph showing the expression level of HIF-1α proteins expressed in BV2 cells after treatment with 100 mM PA. **(E)** Expression of HIF-1α in the CA3 of the hippocampus and cortex of mice in the two groups observed by immunofluorescence (*n* = 3/group). Scale bars = 50 μm.

Then, we evaluated the effect of a hyperlipidaemic diet on the hypoxic environment in the mouse brain. In HFD-fed mice, the protein levels of HIF-1α were significantly increased, as shown by western blotting. BV2 cells treated with PA yielded similar results. To determine the localization of HIF-1α in the two groups, immunofluorescence analysis was performed (Figure 3). HIF-1α was highly expressed, and the overlay images showed that HIF-1α accumulated in the nucleus rather than the cytoplasm in the HFD group (Figure 3E). These results suggested that HIF-1α was increased in hypoxic tissue in the hypoxic environment induced by the hyperlipidaemic diet.

### A high-fat diet increased the expression of brain senescence markers

**FIGURE 4:**
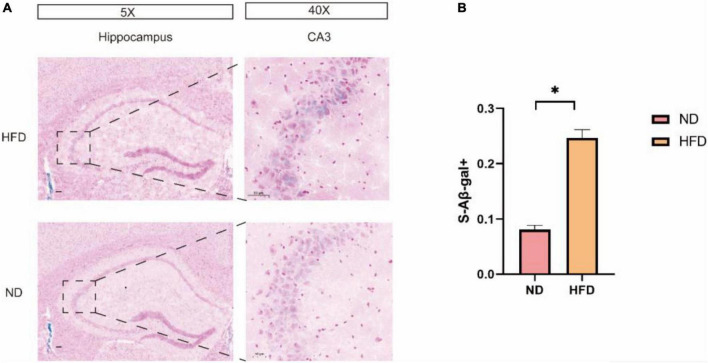
A HFD induced hippocampal cell senescence. **(A) Representative images of SA-**β-Gal**^+^** in HFD mice and ND mice in the hippocampus. The enlarged boxed region (right) shows the region CA3-positive cells. Scale bars = 50 μm. **(B)** Quantification of SA-b-GalC in total cells in the hippocampus of ND and HFD mice (n = 3/group) *p < 0.05.

Senescence-associated β-galactosidase (SA-β-Gal) is a common marker for senescence. SA-β-gal staining of brain tissues was used as a brain senescence marker. Our results show that after 24 weeks of high-fat diet feeding, a significant increase in the number of SA-b-gal-positive cells was demonstrated in the mouse hippocampus when compared to that in the normal diet groups especially in region CA3 (Figure 4).

### A high-fat diet triggers cellular apoptosis in mice brain

**FIGURE 5:**
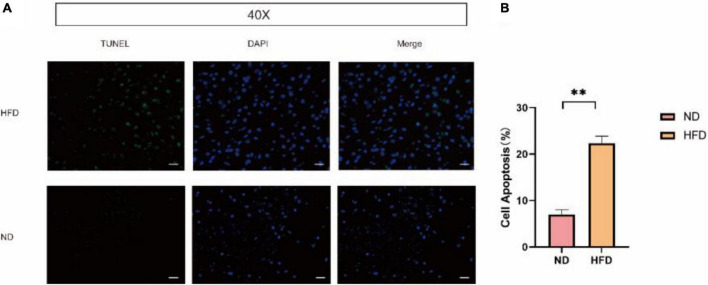
A HFD boosted cell apoptosis in mice brain. **(A)** TUNEL staining was applied to assess the effects of HFD induced on the apoptosis of brain cell. Scale bars = 50 μm. **(B)** Percentage of TUNEL-positive cells by TUNEL staining (n = 3/group) **p < 0.01.

Cellular apoptosis plays numerous important age-related degenerative pathophysiological roles. We also performed terminal deoxyribonucleotide transferase (TdT)-mediated dUTP nick-end labeling (TUNEL) staining shown in Figure 5. After 24 weeks of a high-fat diet, apoptosis was significantly increased in the brains of the HFD group.

### A high-fat diet influences autophagic flux by suppressing the conversion of autophagosomes to autophagolysosomes

Autophagic flux was measured by examining the levels of the autophagy-related proteins LC3, Atg3, and Beclin1 by western blotting. The expression of LC3, Atg3, and Beclin1 was significantly increased; moreover, the expression of the elective autophagy receptor SQSTM1/p62 was also increased, suggesting that a HFD may affect autophagic flux by increasing autophagosome formation. To confirm the role of autophagosomes in the mouse hippocampus, we performed transmission electron microscopy, which is a standard method to examine autophagy activation. Under an electron microscope, the number of autophagosomes was increased in HFD-induced cells, and the number of autophagolysosomes was decreased. Abnormal glial morphology and increased apoptosis in HFD-fed mice were accompanied by mitochondrial depolarization. Figures 8Eb,d shows a significantly abnormal synaptic structure and blurred or missing synaptic clefts. We tested this hypothesis, and the results from our *in vitro* HFD model using BV2 cells was consistent with the *in vivo* results. Western blotting showed that PA treatment significantly increased the expression of LC3II/LC3I, Atg3 and Beclin1 and increased the accumulation of p62 compared with those in the control group. These data suggest that a long-term HFD feeding can suppress autophagic degradation.

**FIGURE 6:**
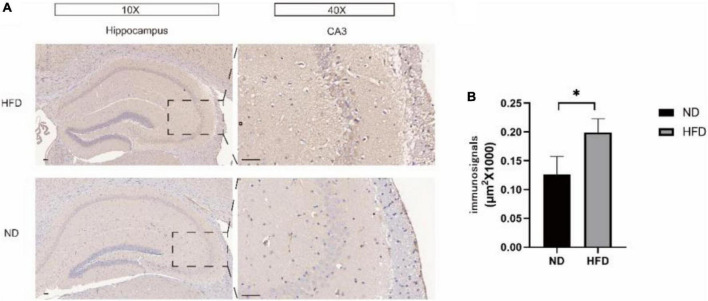
**(A)** Hippocampal immunohistochemical staining using an antibody against Aβ42 was performed to detect the Aβ42 load in hippocampal slices from the ND group and HFD group. The right panel shows representative Aβ42 plaque staining in region CA3. **(B)** Immunohistochemical analysis of Aβ42 protein expression. Significant differences compared with the ND group.

**FIGURE 7:**
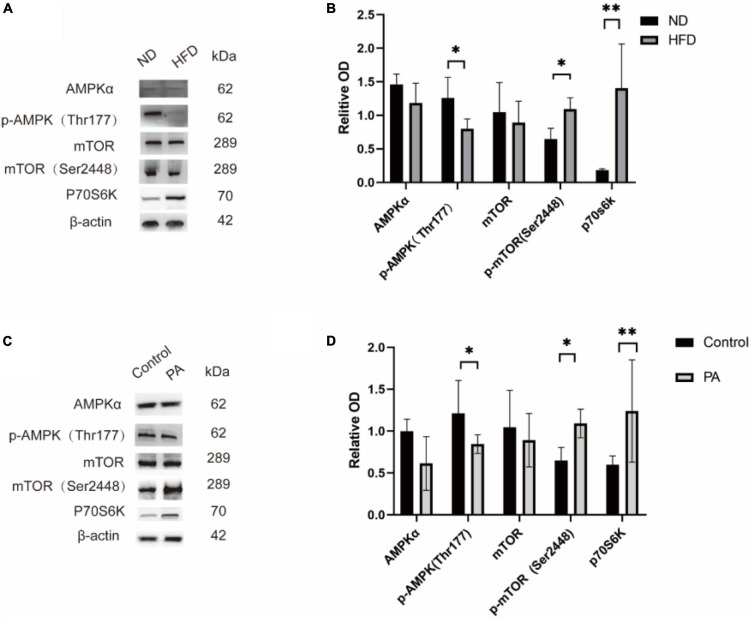
Effect of high-fat diet on AMPK/mTOR/p70S6K pathway protein levels. Representative western blotting bands of mTOR, p-mTOR, AMPK, p-AMPK, and p70S6K in the hippocampus of the ND and HFD groups (shown in panel **A)**. Statistical results showed that a high-fat diet increased p-mTOR levels and the p-mTOR/mTOR ratio, decreased p-AMPK levels and the p-AMPK/AMPK ratio and decreased p70S6K in the hippocampus (ND, *n* **=** 4 mice; HFD, *n* **=** 4 mice) (shown in panel **B)**. BV2 cells treated with were done with PA medium (100 mM), and showed the same result **(C,D)** *p < 0.05, **p < 0.01.

**FIGURE 8:**
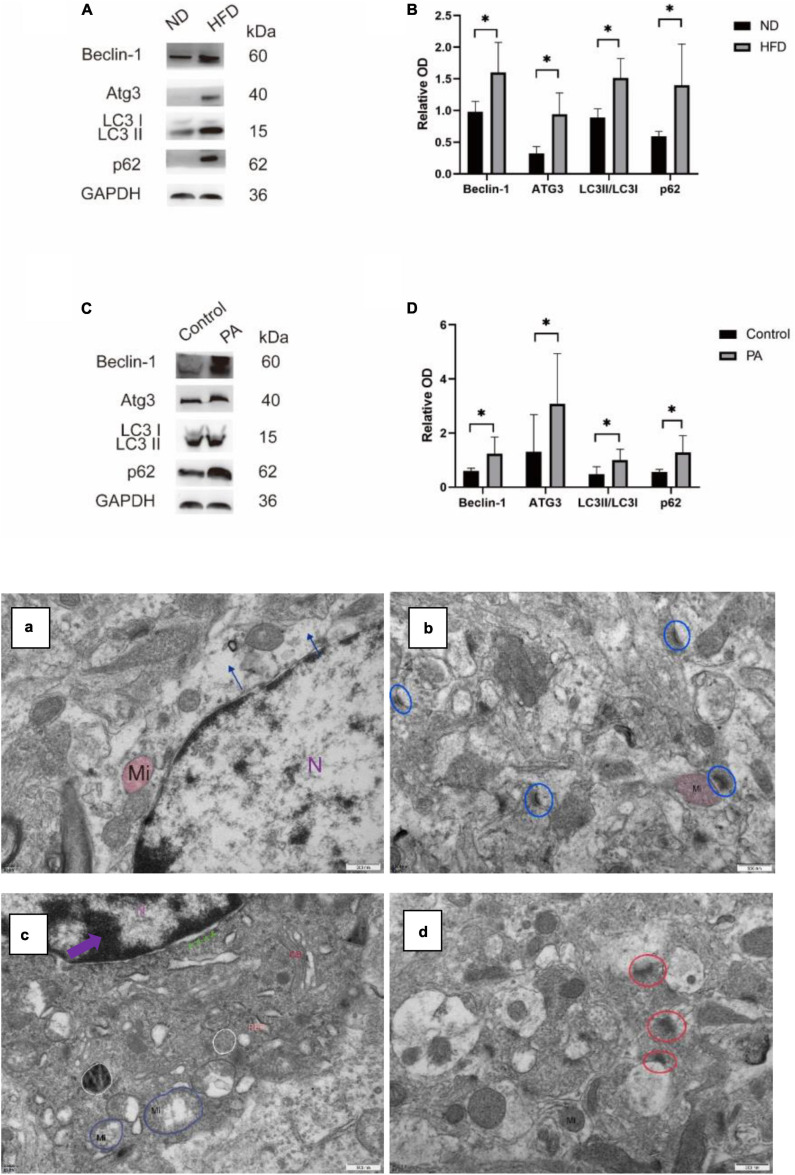
The expression levels of autophagy-associated proteins and the ultrastructure of autophagosomes in the hippocampus of mice under an electron microscope. **(A,B)** Representative western blotting bands of Beclin-1, ATG3, LC3, and P62 in the hippocampus of mice and their quantification. **(C,D)** BV2 cells treated with PA medium (100 mM) showed increased expression of Beclin-1, ATG3, LC3, and P62 compared with the CON group (ND, *n* = 4 mice; HFD, *n* = 4 mice). **(E)** Alterations in hippocampal ultrastructure were examined by transmission electron microscopy. **(a)** The gliocyte lost its cytosol (blue arrow) in ND. N, nucleus; Mi, mitochondria, ×30000. **(b)** The synaptic structure was intact, with normal histological structure in ND (blue circle). **(c)** The nucleus condensed, and the chromosomes gathered to assemble chromatin (purple arrow) and widened the nuclear gap (green arrow) and mitochondrial mild swelling (blue circle) and autophagosomes (white circle) in the HFD group. RER, rough endoplasmic reticulum, GB, Golgi bodies ×30000. **(d)** The structure of presynaptic membrane and synaptic gap is not clear in HFD (red circle) ×30000, scalar bar = 500 μm.

### The AMPK-mTOR-p70S6K signaling pathway, which regulates autophagy, is activated by a high-fat diet

To confirm whether the increase in autophagy induced by HFD was dependent on the regulation of the AMPK-mTOR-p70S6K signaling pathway, C57BL/6J mice were fed a HFD for 6 months. Western blot analysis revealed that the levels of phosphorylated AMPK (Thr172) were decreased, and the levels of phosphorylated mTOR (Ser2448) and p70S6 were decreased, as shown in Figure 7.

### A high-fat diet caused hippocampal amyloidosis to increase in aged C57BL/6 mice

Amyloid-β (Aβ) peptides have been reported to impair synaptic function, long-term synaptic plasticity and memory performance Walsh et al. (2002) and Cleary et al. (2005). We performed immunohistochemistry to detect the expression of Aβ42 in the hippocampus of the ND group and HFD group. Interestingly, as shown in Figure 6, compared with the ND group, the HFD group had significantly increased expression of Aβ42 in the hippocampus, especially in the CA3 region.

## Discussion

**FIGURE 9:**
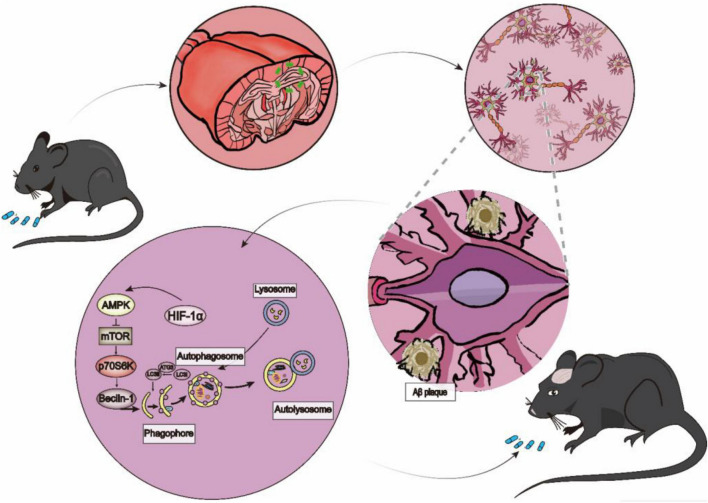
The Process and Regulation of Autophagy. A long-term HFD influenced brain damage by upregulating the expression of HIF-1α, which inhibited autophagy by inhibiting the expression of p-AMPK/p-mTOR/P70S6K and promoting the expression of p62. Meanwhile, HFD increased the protein level of Aβ_42_.

This study aimed to investigate HFD-induced obesity on the ageing process and cognitive or behavioral changes. In this study, we found that 24 weeks of a HFD effectively induced phenotypic changes, including obesity and changes in skin and fur, in mice. In addition, the mice also showed learning and memory deficits. Moreover, the HFD-induced obesity activated tissue hypoxia, upregulated the expression of HIF-1α, and influenced autophagy by decreasing the p-AMPK/AMPK ratio and increasing the p-mTOR/mTOR ratio in the hippocampus. The results of the *in vitro* experiment were consistent with those of the *in vivo* experiment. Overall, our results demonstrated that long-term HFD-induced obesity may increase HIF-1α levels, inhibit AMPK phosphorylation and promote mTOR phosphorylation to inhibit autophagy and increase apoptosis and cellular senescence, which eventually leads to a senescent phenotype and disturbances in cognitive function in mice. Accumulating evidence indicates that chronic HFD feeding can cause obesity, insulin resistance, and glucose intolerance Forouhi et al. (2018). However, research on how brain metabolic manipulations improve the quality of health and thus prolong life in elderly individuals is still in its infancy. Specifically, accelerated ageing and cognitive dysfunction following HFD feeding are poorly understood. Impaired autophagy is involved in neurodegenerative age-related diseases, such as AD, PD, and HD, as well as in lysosomal storage disorders Hansen et al. (2018). It is widely accepted that autophagy is divided into three categories: macroautophagy, molecular chaperone-mediated autophagy and microautophagy. Among them, the most widely studied is macroautophagy. Autophagy is a dynamic process that consists of several steps, including (i) phagosome formation, (ii) autophagosome formation, (iii) lysosome fusion to autophagolysosomes, and (iv) the degradation of autophagolysosomes (Figure 9). Microtubule-associated protein light chain 3β (also known as LC3) is an essential macroautophagy protein that is present from autophagosome formation to fusion with lysosomes. LC3 is divided into two subtypes: soluble LC3-I and lipidated LC3-II. LC3I is first activated by autophagy-related protein 7 (Atg7) and then transferred to Atg3 (a ubiquitin-2-like protein), and the conversion of LC3 from the LC-I-cleaved form to the conjugated form (LC3II) is considered a key step in autophagosome formation Tanaka et al. (2012). Beclin1 is a crucial molecule in autophagy due to its ability to initiate autophagosome formation, mediate the recruitment of autophagy-related proteins and promote the formation and maturation of autophagosomes Choi et al. (2015). The mTOR signaling pathway is well known to be a negative regulator of autophagy. Activated AMPK (p-AMPK, phosphorylated AMPK) inhibits mTOR, resulting in enhanced autophagy. The major downstream target of mTOR is P70S6K. An adverse haemodynamic profile and diminished cerebral blood flow were observed in adipose tissue from obese humans, suggesting a hypoxic state in the tissue, which likely contributes to metabolic dysfunction.

Previous studies have established a link between obesity and an increased burden of senescent cells Tumasian et al. (2021). In addition, Shin et al. showed that an increase in HIF-1α contributes to increased p-tau expression in neurons and astrocytes in the hippocampus, eventually resulting in cognitive impairment Shin et al. (2019). Moreover, it has been previously reported that autophagic flux is increased in the initial phase of hypoxia and later results in impaired autophagy-mediated clearance (Cui et al., 2017). Whereas AMPK depresses the activity of HIF-1α, HIF-1α promotes autophagic flux in an AMPK-independent manner Li et al. (2015). In the present study, our results showed that a HFD markedly induced the protein expression of p-AMPK and reduced the expression of p-mTOR in the mouse hippocampus, which might be due to eutrophication Hu et al. (2018). Emerging evidence suggests direct links between autophagy and Aβ, which may contribute to AD onset or progression Pickford et al. (2008). Normally, Aβ aggregates, can also be degraded by the autophagy-lysosomal pathway (ALP). Defective ALP, which is increased formation of autophagosomes not only leads to Aβ_42_ accumulation, but also hinders its degradation. In addition, it has been shown that defective ALP can overproduce neurovirulent Aβ_40_ and Aβ_42_ Chu et al. (2013).

In summary, our results provide the first evidence that a long-term HFD causes accelerated ageing and cognitive dysfunction compared to a normal diet. Autophagy dysfunction caused by disrupted lipid metabolism and tissue hypoxia in the brain may be associated with dietary fat. In comparison, previous studies in this field mostly studied young mice fed a HFD for a shorter time. Thus, our study revealed that a HFD alters the rate of pathological autophagosomes *in vitro* and *in vivo*. Ageing is characterized by a progressive loss of physiological integrity in various organs throughout life. Dietary nutrients are among the most critical environmental factors that modulate health span and lifespan. However, how a HFD affects brain damage is still unclear. Our findings may provide a means for the diagnosis or personalized treatment of congenital premature ageing syndromes, AD and PD and resolve the challenges in the ageing population. Furthermore, our work provides novel insight into the importance of HFD feeding in midlife in mice.

## Data availability statement

The raw data supporting the conclusions of this article will be made available by the authors, without undue reservation.

## Ethics statement

The animal study was reviewed and approved by the Chongqing General Hospital.

## Author contributions

ZC and FC were responsible for the conception of the idea, data analysis, and manuscript preparation. FC, WY, SW, MY, JW, HL, QZ, and SL performed the experimental work. ZC and FC prepared and revised the manuscript. All authors contributed to the article and approved the submitted version.
